# Personalized High-Resolution Genetic Diagnostics of Prostate Adenocarcinoma Guided by Multiparametric Magnetic Resonance Imaging: Results of a Pilot Study

**DOI:** 10.3390/ijms26125648

**Published:** 2025-06-12

**Authors:** Jacek Wilkosz, Dariusz Wojciech Sobieraj, Tadeusz Kałużewski, Jakub Kaczmarek, Jarosław Szwalski, Michał Bednarek, Agnieszka Morel, Żaneta Kasprzyk, Łukasz Kępczyński, Jordan Sałamunia, Agnieszka Gach, Bogdan Kałużewski

**Affiliations:** 1 Second Department of Urology, Medical University of Lodz, 93-513 Lodz, Poland; 2 Department of General, Oncological, and Functional Urology, Copernicus Memorial Voivodship Multidisciplinary Centre for Oncology and Traumatology, Medical University of Lodz, 93-513 Lodz, Poland; 3 Department of Urology and Urological Oncology, John Paul II Podkarpackie Province Hospital in Krosno, 38-400 Krosno, Poland; 4 Department of Genetics, Polish Mother’s Memorial Hospital Research Institute, 93-338 Lodz, Poland; 5 General Surgery Department—Endoscopy Unit and One-Day Surgery Department (Urology), Bonifraters’ Medical Center, 93-378 Lodz, Poland; 6 CYTOPATH S.A., Histopathological Laboratory, 90-552 Lodz, Poland; 7 Laboratory of Medical Genetics, R&D Division, GENOS Sp. z o.o., 91-033 Lodz, Poland

**Keywords:** prostate cancer, MRI-guided biopsy, next-generation sequencing, molecular profiling, personalized oncology

## Abstract

The upcoming wave of personalized medicine, driven by genomic diagnostics and artificial intelligence, demands clearly defined pre-laboratory and laboratory procedures to ensure the acquisition of DNA and RNA of sufficient quantity and quality. In prostate cancer oncogenetics, diagnostic and prognostic assessments increasingly rely on personalized approaches, including Comprehensive Genomic Profiling (CGP). In this pilot study, we aimed to establish optimal pre-analytical and analytical conditions for selected genetic diagnostic methods using tissue samples acquired through multiparametric MRI-guided biopsy. Tissue specimens from thirteen patients were processed for DNA isolation, fluorescence in situ hybridization (FISH), and next-generation sequencing (NGS). Comparative analyses were performed on DNA derived from both fresh and formalin-fixed, paraffin-embedded (FFPE) samples. Sequencing quality metrics demonstrated markedly superior performance in fresh tissue compared to FFPE. These results highlight the importance of standardized tissue collection and processing protocols to enable reliable molecular diagnostics in prostate cancer. Our findings support the feasibility of integrating high-quality genomic testing into routine biopsy workflows and emphasize the need for further large-scale validation.

## 1. Introduction

In 2023, prostate cancer (PC) was the most frequently diagnosed malignant tumor among men in Poland, accounting for 21.2% of cases. It was also the third-leading cause of cancer-related death among men (10.8%), followed by lung and colorectal cancers [1]. Early, reliable, and multiparametric diagnostics are crucial for designing optimal treatment programs and, consequently, reducing mortality. Total and free prostate-specific antigen (PSA) levels in serum, prostate volume determined by transrectal ultrasound (TRUS), and derived PSA parameters such as PSA density (PSAD) calculated via classification and regression trees (CARTs) are prerequisite indicators for considering invasive diagnostic procedures [2,3]. TRUS-guided transperineal or transrectal biopsy of the prostate combined with magnetic resonance imaging (MRI)/ultrasound (US) fusion biopsy is currently the widely accepted standard for invasive prostate cancer diagnostics [4,5]. The broader implementation of high-frequency microultrasound (29 MHz) [6] and semiautomatic biopsy transfer devices [7] has the potential to significantly improve the sensitivity and specificity of invasive diagnostics, offering new opportunities for clinical diagnoses. Histopathological (HP) examination supported by immunohistochemistry (IHC) remains the gold standard for prostate cancer diagnosis [8]. In addition to HP results, genetic testing has become an increasingly valuable source of information, enabling the personalization of diagnostic and therapeutic processes [9,10]. The most commonly available material for genetic analysis consists of archived paraffin blocks containing the tissue used in HP diagnostics. However, even the most meticulous histopathological processing leads to nucleic acid degradation [11,12]. Several studies have evaluated different DNA isolation techniques, comparing the quality of fresh versus formalin-fixed, paraffin-embedded (FFPE) tissue for diagnostic purposes [13,14,15,16], as well as the efficiency of genomic variant detection using various molecular methods [17,18,19,20]. Whole-genome sequencing analyses have shown that, in FFPE-derived samples, only approximately 50% of the variants identified in matched fresh tissue DNA can be reliably detected [21]. Despite the limitations associated with nucleic acid quality, the risk of obtaining a false-negative result due to the absence of tumor cells is considered greater than the risk related to nucleic acid degradation during FFPE processing [22]. In this study, we presented a method for obtaining MRI-guided prostate biopsy samples that allows for the isolation of high-molecular-weight DNA meeting the qualitative and quantitative criteria required for next-generation sequencing (NGS) and the application of fluorescence in situ hybridization (FISH) at the initial stage of the diagnostic process.

## 2. Results

### 2.1. DNA Quality and Quantitative Metrics

**Figure 1:**
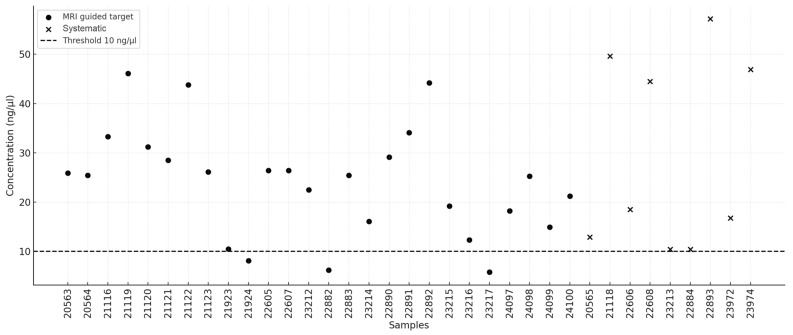
D that we have no conflicts trations.

**Figure 2:**
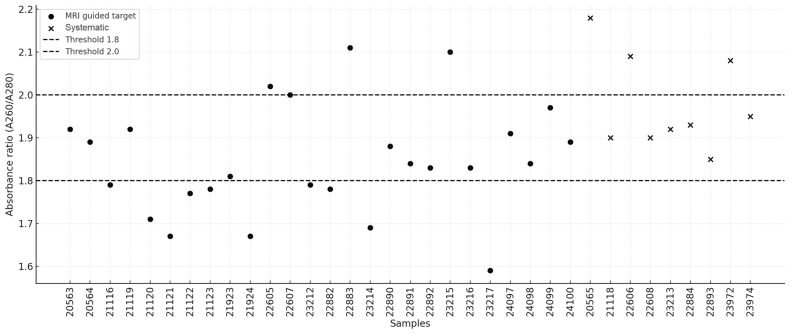
Distribution of sample absorbance ratios.

High-molecular-weight DNA was successfully isolated from all 35 biopsy samples (26 MRI-guided targets and 9 systematic samples), with concentrations ranging from 5.8 ng/μL to 57.2 ng/μL. A summary of the isolation results is presented in Table 1. The following quality criteria were adopted: concentration ≥10 ng/μL and A260/A280 absorbance ratio between 1.8 and 2.0. The mean DNA concentration extracted from MRI-guided targets was 24.08 ng/μL (95% CI: 19.64–28.53), whereas the mean concentration for systematic biopsies was 29.68 ng/μL (95% CI: 14.83–44.53). The median values were 25.4 ng/μL (95% CI: 19.2–27.45) and 18.5 ng/μL (95% CI: 10.4–49.6), respectively. No statistically significant difference was observed between the two groups (U = 105.0, *p* = 0.664). In three MRI-guided target samples (11.54%), the DNA concentration was below the threshold of 10 ng/μL. The distribution of DNA concentrations is illustrated in Figure 1. In terms of the A260/A280 absorbance ratio, the mean values were 1.85 (95% CI: 1.79–1.90) for MRI-guided targets and 1.98 (95% CI: 1.89–2.06) for systematic biopsies, indicating a statistically significant difference (U = 47.0, *p* = 0.0087). The median values were 1.84 (95% CI: 1.79–1.90) and 1.93 (95% CI: 1.9–2.09), respectively. Half of the samples from the MRI-guided targets fell outside the recommended absorbance range of 1.8–2.0, whereas this proportion was one-third for the systematic samples. The distribution of the absorbance ratios is presented in Figure 2.

Additionally, DNA was isolated from four paraffin-embedded tissue blocks prepared for routine histopathological diagnostics. The overall isolation results are presented in Table 2. The mean DNA concentration obtained was 21.85 ng/μL (95% CI: 6.06–37.64), while the absorbance ratio A260/A280 was 1.64 (95% CI: 1.54–1.73). The median values were 19.45 (95% CI: 12.8–35.7) and 1.66 (95% CI: 1.55–1.67), respectively.

**Figure 3:**
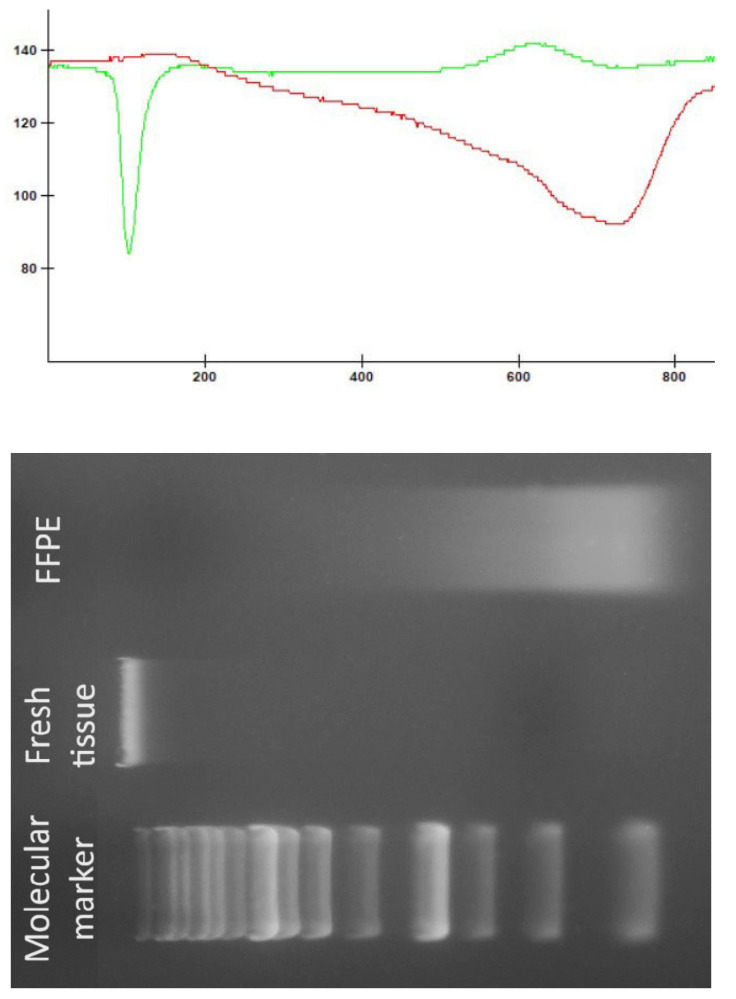
Comparison of DNA quality and integrity between fresh tissue and FFPE samples. The green line represents the densitometric measurements of the DNA sample from fresh tissue, whereas the red line represents the densitometric measurements of the DNA sample obtained from FFPE.

No statistically significant difference was detected in the DNA concentrations obtained from fresh tissue biopsies collected from MRI-guided target locations compared with those obtained from paraffin-embedded tissue blocks (U = 59.0, *p* = 0.692). However, a significant difference was observed in the absorbance ratio (U = 99.0, *p* = 0.00448), favoring samples isolated from fresh tissue. Figure 3 presents a comparison of DNA quality and integrity between fresh tissue and FFPE samples.

### 2.2. Patient Clinical Characteristics

Among the thirteen patients included in the study, four in whom a biopsy was performed via the SmartBx device were selected for cytogenetic and molecular analysis. The following section presents their clinical findings.

#### 2.2.1. Patient 1

At the age of 65, the patient sustained a head injury, resulting in a subdural hematoma in the left cerebral hemisphere. The hematoma was evacuated through a frontoparietotemporal craniotomy. During a routine abdominal ultrasound performed during hospitalization, an excessively distended urinary bladder extending into the mid-abdomen was noted, accompanied by impaired micturition and an enlarged prostate measuring 39 × 51 mm. MRI of the prostate revealed a gland measuring 45 × 56 × 55 mm (AP × LR × SI). In the left lobe, at the mid-gland level within the transitional zone (TZa), a 6 mm area with low signal intensity and diffusion restriction was identified, meeting the PI-RADS v2 criteria for category 3. Two years later, in the same location, the lesion had increased to 10 mm and was classified as PI-RADS v2 category 4. During a three-year posthospitalization follow-up, PSA levels were measured six times, showing a progressive increase from 9.15 ng/mL to 16.62 ng/mL (reference: <4.10 ng/mL). The patient had a 40-year history of smoking approximately half a pack of cigarettes per day. These findings prompted the decision to perform a diagnostic fusion biopsy of the prostate. Histopathological examination of the biopsy sample revealed benign prostatic hyperplasia with focal glandular atrophy and chronic inflammation.

#### 2.2.2. Patient 2

The indication for performing a conventional “blind” prostate biopsy four years ago in a 57-year-old patient was the presence of lower urinary tract symptoms (LUTS) accompanied by persistently elevated PSA levels and a family history of colorectal cancer, along with reported *BRCA2* mutation carrier status (as stated by the patient; no confirming medical documentation was available). The biopsy yielded five oligobiopsies of prostate tissue, revealing small foci of well-differentiated Gleason 6 (3 + 3) prostate cancer. The malignancy was present in one out of five cores, accounting for approximately 5% of the sample’s surface area, with very small tumor foci measuring 2 mm and 1 mm in diameter. IHC analysis revealed AMACR+ and BCC+ staining, with AMACR positivity in the cancer foci. In the following years, 2 additional standard prostate biopsies were performed, yielding 10 oligobiopsies free of cancer infiltration and 22 biopsy samples without atypical proliferation but showing mild glandular atrophy and focal chronic inflammatory infiltration. The initial histopathological findings were subjected to retrospective regrading, which did not confirm the presence of cancer. In one of four additional slides, at the periphery of a core taken from the transition zone, a few suspect glandular structures with marginal AMACR expression and several p63+ cells at the periphery were observed. Two similar glands with comparable morphology were identified in another biopsy sample. Owing to persistent LUTS, indications were established for a transperineal fusion biopsy. A total of 22 oligobiopsies were obtained and subjected to histopathological evaluation. The findings included mild glandular atrophy, focal chronic inflammatory infiltration, and hyperplastic changes. No atypical proliferation was observed in the prostate tissue. IHC analysis revealed AMACR (−) and BCC (+).

#### 2.2.3. Patient 3

A 70-year-old male patient had been under urological care for 20 years due to intermittent polyuria and nocturia (up to five episodes per night) and was managed with tamsulosin. Recently, he reported pain in the right groin. According to the patient, his older brother had undergone radical prostatectomy for prostate cancer at the age of 66. These clinical factors constitute the indications for performing a prostate fusion biopsy. Histopathological examination revealed acinar adenocarcinoma of the prostate (ICD-O 8140/3) with a Gleason score of 3 + 3 = 6 points, corresponding to prognostic Grade Group 1. The tumor exhibited cribriform structures but showed no evidence of neuroinvasion, angioinvasion, or extraprostatic extension. A total of 13 oligobiopsies were analyzed, with two biopsy cores containing tumor infiltration occupying between 1% and 50% of the core length. The Gleason score remained 3 + 3 = 6. IHC analysis revealed AMACR (+) and BCC (−) staining. The patient subsequently underwent radical prostatectomy, followed by adjuvant radiotherapy and chemotherapy. Positron emission tomography (PET) was subsequently performed and revealed a metastatic lesion in the right iliac bone.

#### 2.2.4. Patient 4

A 64-year-old male patient presented with LUTS and persistently elevated PSA levels. MRI revealed a PI-RADS v2 score of 4 in the right lobe and a score of 3 in the left lobe. The patient had a history of smoking one pack of cigarettes per day for 30 years but had quit smoking eight years prior to evaluation. The clinical indications for performing a transperineal fusion biopsy were persistently elevated PSA levels and MRI findings. Histopathological examination of the biopsy specimen confirmed acinar adenocarcinoma of the prostate (ICD-O 8140/3), with immunohistochemical staining showing p63 (−) and AMACR (+). The tumor had a Gleason score of 3 + 3 = 6, corresponding to prognostic Grade Group 1. No evidence of neuroinvasion or angioinvasion was observed. Tumor infiltration was present in one biopsy core, occupying 10% of the tissue length, and in another core, occupying 20%. The patient underwent radical prostatectomy. Postoperative histopathological evaluation confirmed acinar adenocarcinoma (ICD-O 8140/3) with an upgraded grading classification: Gleason score 3 + 4 = 7, corresponding to prognostic Grade Group 2. The percentage of Gleason pattern 4 tumors within Gleason 7 (3 + 4, 4 + 3) tumors was 20%. No tertiary Gleason 5 component (<5%) was identified.

### 2.3. TMPRSS2/ERG Fusion Evaluation

**Figure 4:**
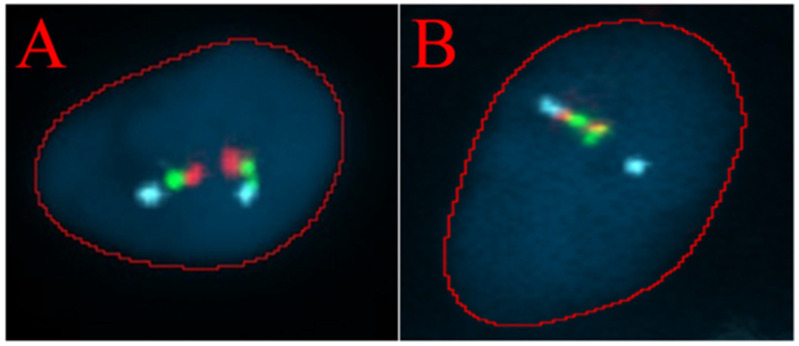
The *TMPRSS2*/*ERG* deletion/breakapart probe is a three-color probe that consists of green probes and red probes positioned on each side of the *TMPRSS2* gene, as well as three blue probes that cover the centromeric region of the *ERG* gene. (**A**) FISH analysis of a single cell showing a normal signal pattern. (**B**) FISH analysis of a single cell showing an abnormal pattern. A blue signal, corresponding to the *ERG* gene, and one combined red and green signal from the derivative chromosome, along with one fused red/green/blue signal from the normal chromosome, are observed.

Analysis of the presence of the *TMPRSS2*/*ERG* fusion gene was performed via FISH. Two positive and two negative results were obtained, which correlated with the histopathological findings (Table 3). Examples of a negative cell and a positive cell are shown in Figure 4.

### 2.4. NGS Results

Next-generation sequencing was performed on six fresh tissue samples and four FFPE samples obtained from four patients. Despite meeting the predefined DNA quality parameters, the sequencing libraries generated from two FFPE samples did not meet the standards required for sequencing. The average sequencing depth achieved was 67× for FFPE samples and 841× for fresh tissue samples. Compared with the fresh tissue samples, the FFPE samples presented a low proportion of aligned reads (mean: 5.98%) and a high duplication rate (mean: 49.37%), where these values were 35.58% and 16.23%, respectively. The detailed quality metrics are presented in Table 4 and Table 5.

**Figure 5:**
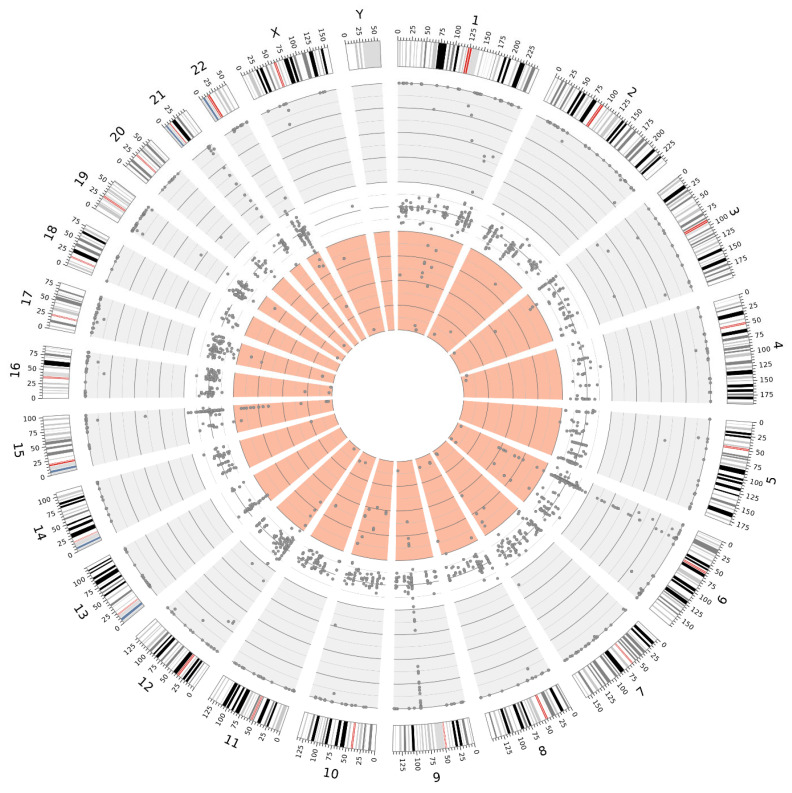
Distribution of variants detected in sample 22890 (fresh tissue), illustrating the distinction between VAF ranges: 100–60% (gray background), 60–40% (white background), and 40–0% (light red background).

**Figure 6:**
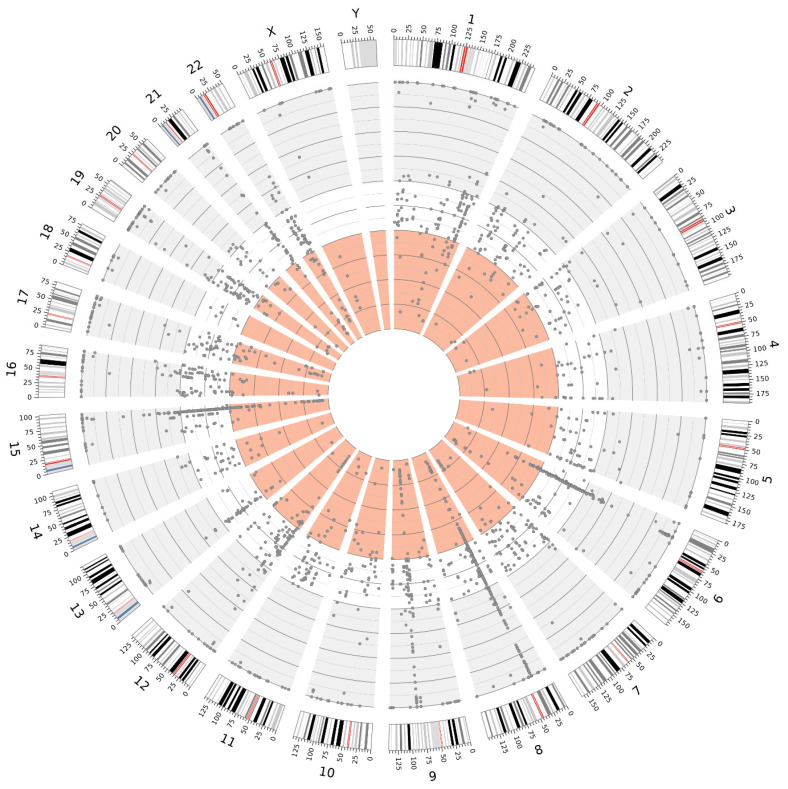
Distribution of variants detected in sample 25487 (FFPE), illustrating the distinction between VAF ranges: 100–60% (gray background), 60–40% (white background), and 40–0% (light red background).

The high sequencing coverage obtained in fresh tissue samples enabled a significantly more accurate estimation of the variant allele frequency (VAF) for individual variants. This, in turn, facilitated the identification of putative germline variants in a homozygous state (VAF~100%) and a heterozygous state (VAF 40–60%). Moreover, fresh tissue samples allowed for a more reliable distinction of low-VAF variants, which may indicate somatic mosaicism, than FFPE samples. The distributions of the variants, exemplified by samples 22890 and 25487 (collected from Patient 4), are illustrated in Figure 5 and Figure 6.

### 2.5. Clinical Follow-Up

**Figure 7:**
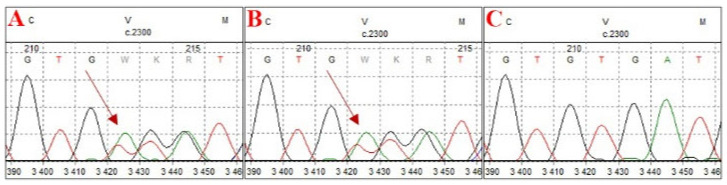
Electrophoregrams from Sanger sequencing confirming the germline origin of the detected variant. (**A**) Sequencing result of the proband, (**B**) sequencing result of the proband’s daughter, confirming carrier status, and (**C**) negative control.

Although the primary aim of the study was diagnostics and the standardization of technical conditions for laboratory procedures, in case 4, a clinically relevant variant, NM_000465.4:c.2300_2301del p.(Val767Aspfs*4), of the *BARD1* gene was identified through NGS analysis. This frameshift variant results in the premature introduction of a stop codon. It is present in control chromosomes in the gnomAD v.4.1.0 database, with a frequency of 0.0000235. The variant has been reported in ClinVar as potentially pathogenic in the context of hereditary cancer predisposition but has not been documented in the COSMIC database. The allele frequency of approximately 50% (340/671 reads) suggests a germline origin. The variant has been classified as pathogenic according to ACMG recommendations for germline variants and as potentially oncogenic on the basis of ClinGen–CGC–VICC guidelines. Pathogenic *BARD1* variants contribute to homologous recombination repair (HRR) deficiency. According to NCCN guidelines, the poly(ADP–ribose) polymerase (PARP) inhibitor olaparib is an effective treatment for metastatic castration-resistant prostate cancer (mCRPC) following prior androgen receptor-directed therapy in patients with HRR gene mutations (particularly mutations in *BRCA1* and *BRCA2*). However, the response of other HRR genes, such as *BARD1*, to olaparib may be variable. The germline origin of the variant was confirmed by Sanger sequencing of DNA isolated from peripheral blood lymphocytes (Figure 7). Following appropriate genetic counseling, the patient’s daughter was tested and identified as a carrier. This finding enables personalized breast cancer risk assessment and the proposal of an appropriate prophylactic strategy.

## 3. Discussion

The collection of biological material during fusion biopsy and the application of appropriate genetic analyses are gaining increasing diagnostic and prognostic significance in the context of prostate cancer. In this study, by optimizing pre-laboratory and laboratory conditions, we demonstrated that high-quality DNA, which is suitable for next-generation sequencing and fluorescence in situ hybridization analysis, can be reliably obtained from MRI-guided biopsies. The extraction of high-molecular-weight DNA from fresh biopsy samples and FFPE samples met the qualitative and quantitative requirements for advanced sequencing applications. Despite the variability in DNA yield and purity, the overall quality parameters remained within an acceptable range for genomic profiling. However, NGS analysis showed significantly better results in fresh tissues than in FFPE samples, particularly in detecting low-allele frequency variants crucial for oncogenetic assessment. Although a direct comparison of detected variants between fresh and FFPE-derived DNA could provide additional insights, the collection of material from different anatomical regions of the prostate introduces biological heterogeneity and precludes definitive conclusions regarding the technical impact of sample preservation methods on variant detection. Moreover, the higher number of low-frequency variants observed in FFPE samples—likely representing sequencing artifacts—could not be confirmed as false positives without matched high-coverage controls. Nevertheless, despite these limitations, objective sequencing quality metrics still clearly demonstrate the technical advantages of fresh tissue over FFPE material. The exposure of prostate biopsy samples to fixation in 10% formalin, for a period that is often difficult to determine precisely but is usually too long, can lead to disintegration of the DNA structure, significantly degrading its quality or even making NGS analysis impossible. Previous studies have evaluated the feasibility of DNA sequencing in prostate cancer and other malignancies by comparing fresh-frozen and FFPE tissue [13,19], reporting results that are consistent with our observations. One major challenge associated with the use of fresh tissue is the lack of prior histopathological assessment. As a result, current clinical guidelines recommend performing molecular testing only after histopathological verification [22]. However, by utilizing MRI and US image fusion, it seems to be possible to obtain precisely located biopsy samples and separate them for simultaneous genetic and histopathological diagnostics. Additionally, the use of semiautomated biopsy core transfer onto a histopathology cassette enables the determination of the biopsy needle insertion direction, precise localization within the prostate structure, and reliable macroscopic assessment. Evaluating the proportion of the cellular component relative to the stromal component in a biopsy core facilitates the selection of a sample that ensures improved DNA isolation outcomes. In the example presented in Figure 1 (picture B), the upper biopsy yields a satisfactory DNA concentration, whereas the lower biopsy does not provide the same level of certainty in achieving the desired DNA yield. If molecular changes characteristic of prostate cancer, such as structural alterations in the *TMPRSS2* and *ERG* genes [23,24], can be identified, histopathological evaluation could be conducted independently. However, it is necessary to develop a genetic diagnostic framework covering all prostate cancer types with sufficiently high sensitivity and specificity. The implementation of high-throughput genetic diagnostics at an early stage of prostate cancer assessment would not only significantly shorten the overall diagnostic process by identifying potential predictive, prognostic, and targeted therapy factors but also provide valuable information on hereditary predispositions, as demonstrated in our study by the detection of a germline *BARD1* pathogenic variant. Genetic testing results could also serve as crucial support for histopathological assessment, for example, by identifying variants characteristic of specific cancer subtypes. It is important to emphasize that this was a small-scale pilot study primarily aimed at assessing the feasibility and technical aspects of using MRI-guided biopsy material for molecular diagnostics. As such, the findings should be interpreted with caution and not be generalized beyond the specific context and scope of this exploratory analysis. Future studies should focus on validating these methodologies in larger cohorts to determine their clinical utility and impact diagnostic and therapeutic decision making. In particular, comparative studies evaluating the detection rates of clinically relevant genomic aberrations in fresh versus FFPE samples are warranted. Such research would help define the true diagnostic advantages of fresh tissue, not only in terms of technical sequencing parameters but also with regard to clinically actionable findings. In the coming years, the quantity and diversity of information available to urologists making diagnostic and therapeutic decisions are expected to increase significantly, potentially posing new challenges in clinical decision making. In this context, the implementation of machine learning and artificial intelligence techniques should be considered supportive tools for analyzing complex diagnostic data while ensuring that the final decision and responsibility remain in the hands of the specialist physician.

## 4. Materials and Methods

### 4.1. Material Acquisition

**Figure 8:**
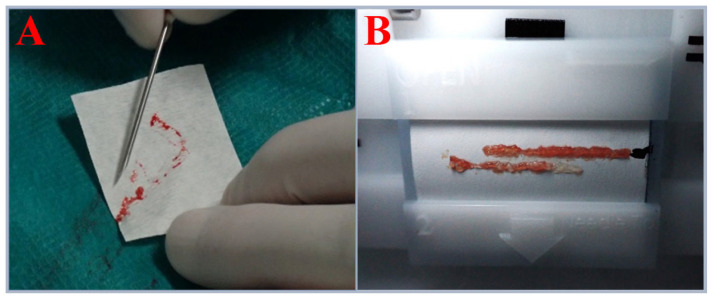
(**A**) Biopsy core acquired using the standard approach. (**B**) Biopsy cores acquired using the SmartBx device.

The study included 35 tissue samples collected from three urological departments over a 24-month period. Only samples obtained from 13 patients eligible for fusion biopsy due to suspected PC, which were not used in routine histopathological diagnostics for technical reasons, were included in the analysis. Prior to the biopsy procedure, MRI/US image fusion was performed via the Koelis system [25]. After 3D reconstruction, both targeted and systematic biopsies were performed in accordance with medical practices best suited for histopathological diagnostics, prioritizing prostate regions with the highest PI-RADS v2 scores. Prostate biopsies were conducted using the BARD BIOPSY SYSTEMS Biopsy Gun (18 G × 25 cm; penetration depth: 22 mm) (Bard Peripheral Vascular, Inc., Tempe, AR, USA). In four cases, biopsy cores were semiautomatically transferred onto histopathology cassette membranes using the SmartBx device (UC-Care Medical Systems, Yokneam, Israel). In the remaining nine cases, the specimens were manually transferred following standard biopsy procedures (Figure 8). Biopsy samples were preserved in RNA Save solution (Biological Industries, Kibbutz Beit Haemek, Israel) at +8 °C and subjected to DNA isolation within 48 h. A total of 35 biopsy cores were collected. Four of the thirteen patients in whom the biopsy was performed using the SmartBx device were selected for FISH and molecular genetic testing, as this method ensured optimal tissue acquisition and integrity, increasing the likelihood of successful processing.

### 4.2. Fluorescence In Situ Hybridization

After routine histopathological diagnostics, paraffin-embedded tissue sections with a thickness of 3 μm were obtained for FISH analysis. These sections were applied to Superfrost Plus™ Gold Adhesion Microscope Slides (Epredia, Portsmouth, NH, USA) following standard procedures. A commercial *TMPRSS2*/*ERG* deletion/breakapart molecular probe (Cytocell, Cambridge, UK) was used in the analysis, in accordance with the manufacturer’s instructions.

### 4.3. DNA Isolation

DNA isolation was performed automatically via the QIAcube Connect MDx instrument (QIAGEN N.V., Hilden, Germany), with the QIAamp DNA Blood Mini Kit for peripheral blood samples (QIAGEN N.V., Hilden, Germany), the DNeasy Blood and Tissue Kit (QIAGEN N.V., Hilden, Germany) for fresh tissue samples, and the QIAamp DNA FFPE Advanced Kit (QIAGEN N.V., Hilden, Germany) for archived material, following the manufacturers’ protocols. The isolation was conducted using biopsy material from regions with PI-RADS v2 scores of 3 or 4, as well as from systematic biopsies. The quality of the DNA samples was assessed spectrophotometrically using the NanoDrop 2000 (Thermo Scientific, Waltham, MA, USA) and electrophoretically in a 1% agarose gel, followed by densitometric analysis. Electrophoretic analysis was carried out using specialized equipment and the complementary gel densitometry software GelScan KTE (BioVectis Inc., Warsaw, Poland).

### 4.4. Next-Generation Sequencing

Next-generation sequencing (NGS) was performed using a 101 bp paired-end method on the NovaSeq 6000 platform (Illumina, San Diego, CA, USA), achieving an average coverage of 842× for fresh tissue samples and 67× for FFPE samples. A panel of 753 cancer-related genes, which is based on the current COSMIC Cancer Gene Census [26], was implemented. Demultiplexing of the sequencing reads was performed via Illumina bcl2fastq (version 2.20). If the sequencing output for a given sample exceeded the requested amount, the reads were downsampled to at least 20% above the required threshold. Adapter sequences were trimmed using Skewer (version 0.2.2) [27]. Sequencing data analysis was conducted via the Illumina DRAGEN platform (version 4.2.4). The reads were aligned to the hg19 reference genome, and duplicate reads were marked. The quality metrics were generated using MultiQC (version 1.22.2). Small variant calling was performed using default parameters. Variant annotation was carried out with Franklin software (Genoox, Tel Aviv, Israel), and variant classification followed the AMP/ACMG and ClinGen–CGC–VICC guidelines. A graphical representation of the variant distribution was generated with Circos v.0.69.9 software [28].

### 4.5. Sanger Sequencing

Sanger sequencing was employed as a validation method to confirm the germline origin of the detected variants. Primers were designed to hybridize with DNA regions encompassing the specified variants via Primer3 input software (version 4.1.0). PCR amplification was performed using StartWarm HS–PCR Mix (A&A Biotechnology, Gdansk, Poland). Following amplification, the PCR products were purified prior to sequencing. A BigDye Terminator v3.1 Cycle Sequencing Kit (Thermo Fisher Scientific, Waltham, MA, USA) was used for sequencing, which was carried out on a 3100 Genetic Analyzer (Thermo Fisher Scientific, MA, USA).

### 4.6. Statistical Analysis

Comparisons were performed using the Mann–Whitney U test. A *p*-value < 0.05 was considered statistically significant. Data were expressed as 95% confidence intervals (95% CI). Statistical analysis was conducted using Python v.3.11 with the SciPy library.

## 5. Conclusions

This study demonstrates that high-quality genetic material suitable for advanced molecular analyses can be successfully obtained from MRI-guided biopsies, enabling comprehensive oncogenetic diagnostics in prostate cancer. The superior performance of NGS in single fresh biopsy core, compared with FFPE material, highlights the importance of optimizing sample collection and processing techniques. The integration of genetic testing into early diagnostic workflows has the potential to refine risk stratification, improve prognostic assessments, and guide personalized treatment strategies. Given the pilot nature of this study, further research involving larger patient cohorts is necessary to validate these findings and fully assess their clinical applicability and impact on patient management.

## Figures and Tables

**Table 1:** Summary of metrics for DNA isolated from fresh tissue samples.

*n*	Sample	Site	Concentration (ng/μL)	Absorbance Ratio (A260/A280)
1	20563	MRI-guided target	25.9	1.92
2	20564	MRI-guided target	25.4	1.89
3	20565	systematic	12.9	2.18
4	21116	MRI-guided target	33.3	1.79
5	21118	systematic	49.6	1.9
6	21119	MRI-guided target	46.1	1.92
7	21120	MRI-guided target	31.2	1.71
8	21121	MRI-guided target	28.5	1.67
9	21122	MRI-guided target	43.8	1.77
10	21123	MRI-guided target	26.1	1.78
11	21923	MRI-guided target	10.5	1.81
12	21924	MRI-guided target	8.1	1.67
13	22605	MRI-guided target	26.4	2.02
14	22606	systematic	18.5	2.09
15	22607	MRI-guided target	26.4	2
16	22608	systematic	44.5	1.9
17	23212	MRI-guided target	22.5	1.79
18	22882	MRI-guided target	6.2	1.78
19	22883	MRI-guided target	25.4	2.11
20	23213	systematic	10.4	1.92
21	22884	systematic	10.4	1.93
22	23214	MRI-guided target	16.1	1.69
23	22890	MRI-guided target	29.1	1.88
24	22891	MRI-guided target	34.1	1.84
25	22892	MRI-guided target	44.2	1.83
26	22893	systematic	57.2	1.85
27	23215	MRI-guided target	19.2	2.1
28	23216	MRI-guided target	12.3	1.83
29	23217	MRI-guided target	5.8	1.59
30	24097	MRI-guided target	18.19	1.91
31	24098	MRI-guided target	25.24	1.84
32	23972	systematic	16.76	2.08
33	24099	MRI-guided target	14.93	1.97
34	24100	MRI-guided target	21.24	1.89
35	23974	systematic	46.88	1.95

**Table 2:** Summary of metrics for DNA isolated from FFPE samples.

*n*	Sample	PI-RADS v2	Concentration (ng/μL)	Absorbance Ratio (A260/A280)
1	25484	4	12.8	1.55
2	25485	4	21.7	1.65
3	25486	3	17.2	1.67
4	25487	4	35.7	1.67

**Table 3:** Detailed results of *TMPRSS2*/*ERG* fusion evaluation.

Case	PI-RADS v2	FISH Result	% of Cells with Fusion	HP
1	4	nuc ish (TMPRSS2,TMPRSS2,ERG)x2[50]	-	benign prostatic hyperplasia, focal glandular atrophy, and chronic inflammation
2	4	nuc ish (TMPRSS2,TMPRSS2,ERG)x2[50]	-	mild glandular atrophy, focal chronic inflammatory infiltration, and hyperplastic changes
3	3	nuc ish (TMPRSS2,ERG)x2(TMPRSS2/TMPRSS2 sep ERG)x1[32/50]	64%	prostatic acinar adenocarcinoma
4	4	nuc ish (TMPRSS2,ERG)x2(TMPRSS2/TMPRSS2 sep ERG)x1[42/50]	84%	prostatic acinar adenocarcinoma

**Table 4:** Quality metrics of NGS of fresh tissue samples.

*n*	Sample	Aligned Reads (on Target)	Proportion of Aligned Reads (in %)	Duplicate Rate (in %)	Average Coverage	Number of Small Variants
1	22890	34.245	36.12	16.81	845.58	2636
2	23215	36.773	38.62	16.8	909.18	2609
3	24097	33.628	34.77	15.24	832.98	2577
4	24098	34.148	35.33	15.18	846.9	2585
5	24099	32.563	33.81	15.58	806.42	2546
6	24100	32.599	34.84	17.78	810.53	2535

**Table 5:** Quality metrics of NGS of FFPE samples.

*n*	Sample	Aligned Reads (on Target)	Proportion of Aligned Reads (in %)	Duplicate Rate (in %)	Average Coverage	Number of Small Variants
1	25486	2.723	6.08	49.63	60.68	2939
2	25487	3.175	5.88	49.11	72.6	2933

## Data Availability

The data and materials used in this study are available from the corresponding authors upon reasonable request.

## List of Abbreviations

CGP	Comprehensive Genomic Profiling
CNV	Copy Number Variation
FISH	Fluorescence In Situ Hybridization
FFPE	Formalin-Fixed Paraffin-Embedded
HP	Histopathology
HRD	Homologous Recombination Deficiency
ICD-O	International Classification of Diseases for Oncology
IHC	Immunohistochemistry
LUTS	Lower Urinary Tract Symptoms
MRI	Magnetic Resonance Imaging
mCRPC	Metastatic Castration-Resistant Prostate Cancer
NGS	Next-Generation Sequencing
PARP	Poly(ADP-ribose) Polymerase
PC	Prostate Cancer
PI-RADS	Prostate Imaging Reporting and Data System
PSA	Prostate-Specific Antigen
PSAD	Prostate-Specific Antigen Density
SNV	Single Nucleotide Variant
SV	Structural Variant
TMB	Tumor Mutational Burden
TRUS	Transrectal Ultrasound
US	Ultrasound
VAF	Variant Allele Frequency
